# Effects of carbon concentration on high-hardness plasma-polymer-fluorocarbon film deposited by mid-range frequency sputtering

**DOI:** 10.1038/s41598-019-46993-0

**Published:** 2019-07-23

**Authors:** Sung Hyun Kim, Mac Kim, Min Seop Um, Woo Jin Choi, Jae Heung Lee, Yong Suk Yang, Sang-Jin Lee

**Affiliations:** 1 Chemical Materials Solutions Center, Korea Research Institute of Chemical Technology, Daejeon, 34114 Korea; 2 Department of Nano Fusion Technology, Pusan National University, Busan, 46241 Korea

**Keywords:** Surfaces, interfaces and thin films, Structural properties

## Abstract

We propose a method for fabricating high-hardness plasma-polymer-fluorocarbon (PPFC) thin films with controllable optical and surface properties via manipulation of the target composition design and sputtering power density. The carbon/polytetrafluoroethylene (PTFE) composite polymeric material targets with the low electrical resistance were prepared by press-molding using a mechanically mixed powder of PTFE, carbon nanotubes, and graphite. The composite targets showed electrical sheet resistances of 0.1–100 Ω/sq. PPFC thin films were deposited by mid-range frequency (MF) sputtering at power densities within 0.62~4.92 W/cm^2^. The maximum surface hardness of the PPFC thin film was 4.75 GPa, which was 21.6 times higher than that of fluorocarbon thin film sputtered from PTFE under the same conditions. With the increase of the carbon concentration in the target, the carbon cross-linking density of the PPFC thin film increased but the fluorine concentration decreased. The concentration of fluorine in the PPFC thin films grew with increasing sputtering power density. The MF sputtered carbon-rich PPFC thin films are controllable with physical properties of optical transmittance, surface hardness and surface water repellency which could be applied as protective layers for transparent flexible devices.

## Introduction

Plasma-polymer-fluorocarbons (PPFC) are fluorinated polymers such as polytetrafluoroethylene (PTFE) or fluorinated organic gases like CF_4_ or C_2_F_6_ that are formed through glow discharge^1^. The Biederman group^2,3^, the Faupel group^4,5^ and the Iwamori group^6,7^ have extensively studied sputtered PPFC thin films and reported optical and mechanical properties of PPFC thin films. Sputtered PPFC thin films can exhibit attractive properties such as high transparency^7^, electrical insulation^8^, superhydrophobicity^9–11^, oleophobicity^12^, and high surface hardness^13,14^; they are suitable for use in display panels, automobiles, and textile fibers.

In foldable and rollable flexible displays, transparent layers capable of protecting display surfaces are indispensable^15,16^; plasma polymer thin films are suitable candidates for such layers. Amorphous carbon, or diamond like carbon (DLC), is a representative plasma polymer material with excellent surface protection properties, having the surface hardness of 20 GPa. As the density of carbon cross-linking, constituting the backbone matrix of the plasma polymer, is increased, the thin film increases in surface hardness^1,17^. However, DLC is high in brittleness and difficult to apply as a flexible protective layer with a small radius of curvature, as well as having low optical transmittance; these properties limit its applicability in transparent flexible devices. In order to overcome these problems, DLC films doped with fluorine and boron have been reported with improved surface characteristics^18,19^. PPFC thin films prepared by sputtering using a fluorine-containing polymer such as PTFE are among the candidates for flexible protective layers to overcome the disadvantages of DLC. PPFC thin films have high optical transmittance and can resist surface contamination by self-cleaning effects^20^. However, the surface hardness of PPFC thin films is low as 0.5–1.0 GPa, similar to that of human fingernails (~0.4 GPa)^21^.

In a previous work, we presented a novel method for PPFC thin film fabrication using carbon nanotube (CNT)/PTFE composite targets, which could use a mid-range frequency (MF) power source, whereas conventional polymer targets can only be sputtered with 13.56 MHz radio frequency power sources, with reflected noise 10^4^–10^5^ times higher than that with a 40 kHz MF power system^22^. In this study, the effect of carbon content on PPFC thin films was researched by increasing the composition ratio of carbon in the sputtering target. The influence of the sputtering power density was also investigated. For a CNT/PTFE composite target using CNT powder with more than 20 wt% CNT, powder dispersion hardly occurs. Therefore, graphite was used as the carbon material. However, the graphite/PTFE composite targets with 5 and 10 wt% graphite showed sheet resistances of 5.98 × 10^14^ Ω/sq and 1.71 × 10^10^ Ω/sq, respectively, unsuitable for MF sputtering. Because of the limited mixing characteristics of CNTs and graphite powder, composite targets with different composition ratios were fabricated. The composite targets were used for MF sputtering at power densities of 2.46–4.92 W/cm^2^ to fabricate carbon-rich PPFC thin films. The structural, surface, and optical properties of the thin films were compared and analyzed according to the carbon concentration of the target and the sputtering power density.

## Results

**Figure 1:**
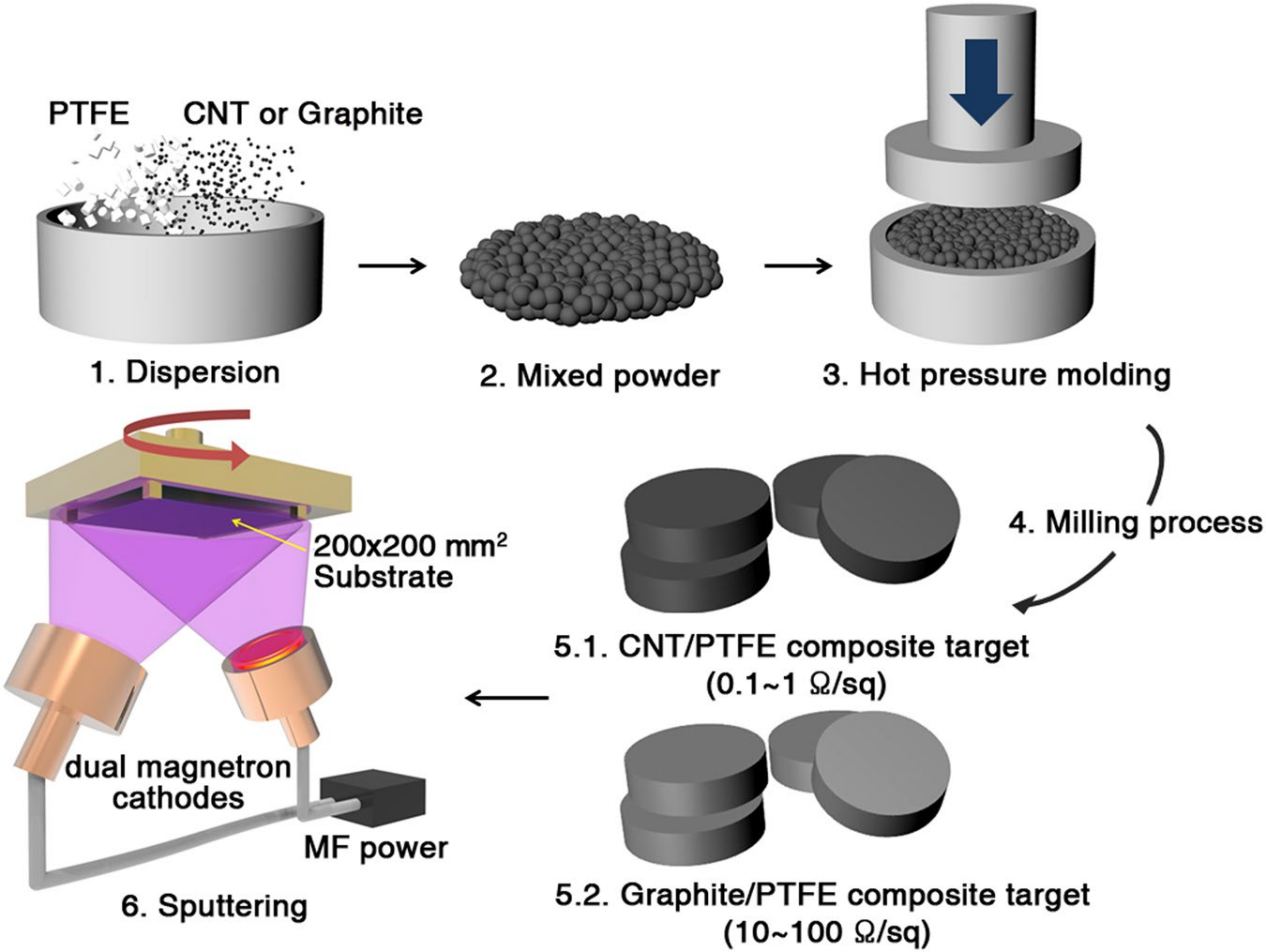
Schematic of CNT/PTFE and Graphite/PTFE composite target production process and the MF sputtering system used for PPFC film deposition.

We mixed CNT powder and PTFE powder in weight ratios of 5:85, 10:90, and 15:85. Graphite powder and PTFE powder were also mixed at weight ratios of 5:85, 10:90, 15:85, and 30:70. Then, the conductive CNT/PTFE and graphite/PTFE targets were shaped into radius 5.3 mm and thickness 6 mm disk. The polymer composite targets were named CNT 5, CNT 10, CNT 15, Graphite 5, Graphite 10, Graphite 15, and Graphite 30. The sheet resistance of the composite targets are reported in Fig. S1. For comparison, pure PTFE without any carbon material was also press-molded to produce a target named Pristine PTFE. The sputtered fluorocarbon films using the fabricated composite targets are named PPFC C5 (CNT 5), PPFC C10 (CNT 10), PPFC C15 (CNT 15), PPFC G15 (Graphite 15), and PPFC G30 (Graphite 30). Figure 1 is a schematic of the composite target production process and the MF sputtering system used for PPFC thin film deposition. The pristine PTFE target was sputtered using an RF power source because of the excessively high sheet resistance of 9.49 × 10^14^ Ω/sq; plasma was not generated for lower frequencies. The sputtered pristine fluorocarbon film was named PPFC 0. We prepared the vacuum chamber with a rotatable substrate size of 200 × 200 mm^2^ and target-to-substrate distance of 240 mm. Base pressure of vacuum system was 6 × 10^−4^ Pa and the processing atmosphere was 0.93 Pa. CNT 5, CNT 10, CNT 15, Graphite 15 and Graphite 30 composite targets were sputtered with a 40 kHz MF power source with dual magnetron cathodes. Pristine PTFE was sputtered with a 13.56 MHz RF power. PPFC thin films were deposited at power densities within 0.62~4.92 W/cm^2^ on polyethylene terephthalate (PET) films and a silicon substrates.

**Figure 2:**
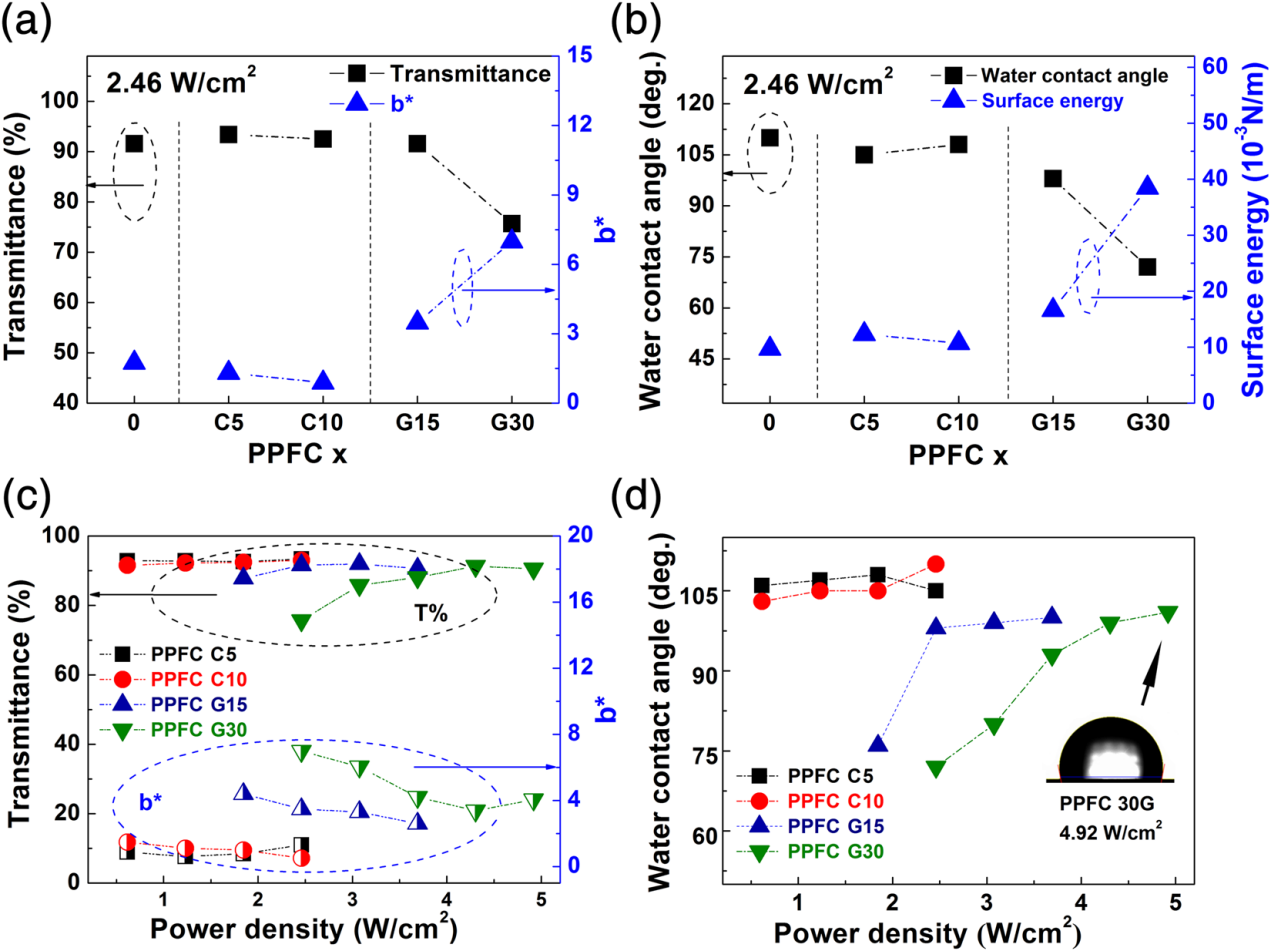
(**a**) 550 nm-optical transmittances and b* yellowness parameters and (**b**) water contact angles and surface energies of PPFC thin films according to carbon concentration in composite polymer target. (**c**) 550-nm optical transmittance and b* yellowness parameters and (**d**) water contact angles of PPFC thin films according to sputtering power density.

Figure 2 shows the optical transmittances, b* values indicating yellowness, and the water contact angles of the PPFC thin films according to the carbon concentration in the targets and sputtering power densities. The optical and surface properties of the PPFC G15 thin films were similar to those of PPFC C15 (Fig. S2). Based on these results, the PPFC 0, PPFC C5, PPFC C10, PPFC G15, and PPFC G30 thin films were used to study the physical properties of the PPFC thin films as functions of the carbon composition. Figure 2(a) shows the 550-nm optical transmittance and b* yellowness parameters of the sputtered PPFC thin films according to the carbon concentrations in the targets. The Carbon/PTFE and Pristine PTFE composite targets were sputtered at the same power density of 2.46 W/cm^2^. PPFC 0, PPFC C5, PPFC C10, and PPFC G15 films show high light transmittances >90%, while the PPFC G30 films have the relatively low light transmittance of 75.70%. The b* values of the PPFC C5 and PPFC C10 films are similar to that of PPFC 0, but they are 3.48 and 6.99 for the PPFC G15 and PPFC G30 films, respectively, up to 5 times higher than that of the PPFC 0 film, due to the composition ratio of CNT and graphite in composite target increased, the amount of sputtered carbon atoms increased relatively. Previous studies have reported that the optical band gap of hydrocarbon materials, including DLC and fluorocarbon, decreases as the pi-states bonding of the sp^2^ hybrid configuration increases^23,24^. The decrease in optical transmittance and the increase in b* values mean an increase in the carbon composition ratio of PPFC thin films. Figure 2(b) shows the water contact angles of the sputtered fluorocarbon film surfaces according to the carbon concentration in the targets and the surface energies calculated by GGFY (Girifalco–Good–Fowkes–Young) method. The surface energy of PPFC C5 is 12.34 × 10^−3^ N/m; that of PPFC C10 was 10.73 × 10^−3^ N/m, similar to that of 9.73 × 10^−3^ N/m shown by PPFC 0. The surface energy increases from 10.0 × 10^−3^ N/m in PPFC 0 to 38.5 × 10^−3^ N/m in PPFC G30. This property change is also attributed to the increase in carbon concentration of the PPFC thin film as the carbon ratio of the composite target is increased. Figure 2(c) shows the 550-nm optical transmittance and b* yellowness parameters of the sputtered fluorocarbon films at different sputtering power densities. The PPFC C5 and PPFC C10 thin films show no significant changes in properties with different sputtering power densities; however, for carbon contents exceeding 15 wt%, the PPFC G15 and PPFC G30 films increase in transparency and hydrophobicity. As the sputtering power density is increased, the light transmittance increases from 87.80% to 91.95% for the PPFC G15 thin film and from 75.70% to 91.32% for the PPFC G30 thin film; b* decreases from 4.40 to 2.60 for PPFC G15 and from 6.99 to 3.38 for PPFC G30. Previous studies of polymer materials using mass spectroscopy analysis have revealed that heavier fragments are sputtered at higher sputtering power densities^25,26^. A previous work also reported that in PTFE decomposition by glow discharge, C, C_2_F_4_, C_2_F_6_, C_3_F_6_, C_3_F_8_, and additional heavier substances are generated^27^. Other researchers have reported that the partial sputter yield of fluorine increases as the impact energy of argon ions is increased^28^. Figure 2(d) shows the water contact angles according to sputtering power density. The changes in the water contact angle of the PPFC thin films almost coincide with the tendency of the changes in light transmittance shown in Fig. 2(c). As the sputtering power density is increased, the PPFC G15 thin film contact angle increases from 76° to 100° and that of the PPFC G30 thin film increases from 72° to 101°. In order to investigate the compositional changes of the thin films with respect to the sputtering power density in this study, the binding energies between carbon and fluorine in the thin films were analyzed by XPS and the quantitative fluorine–carbon ratios were calculated.

**Figure 3:**
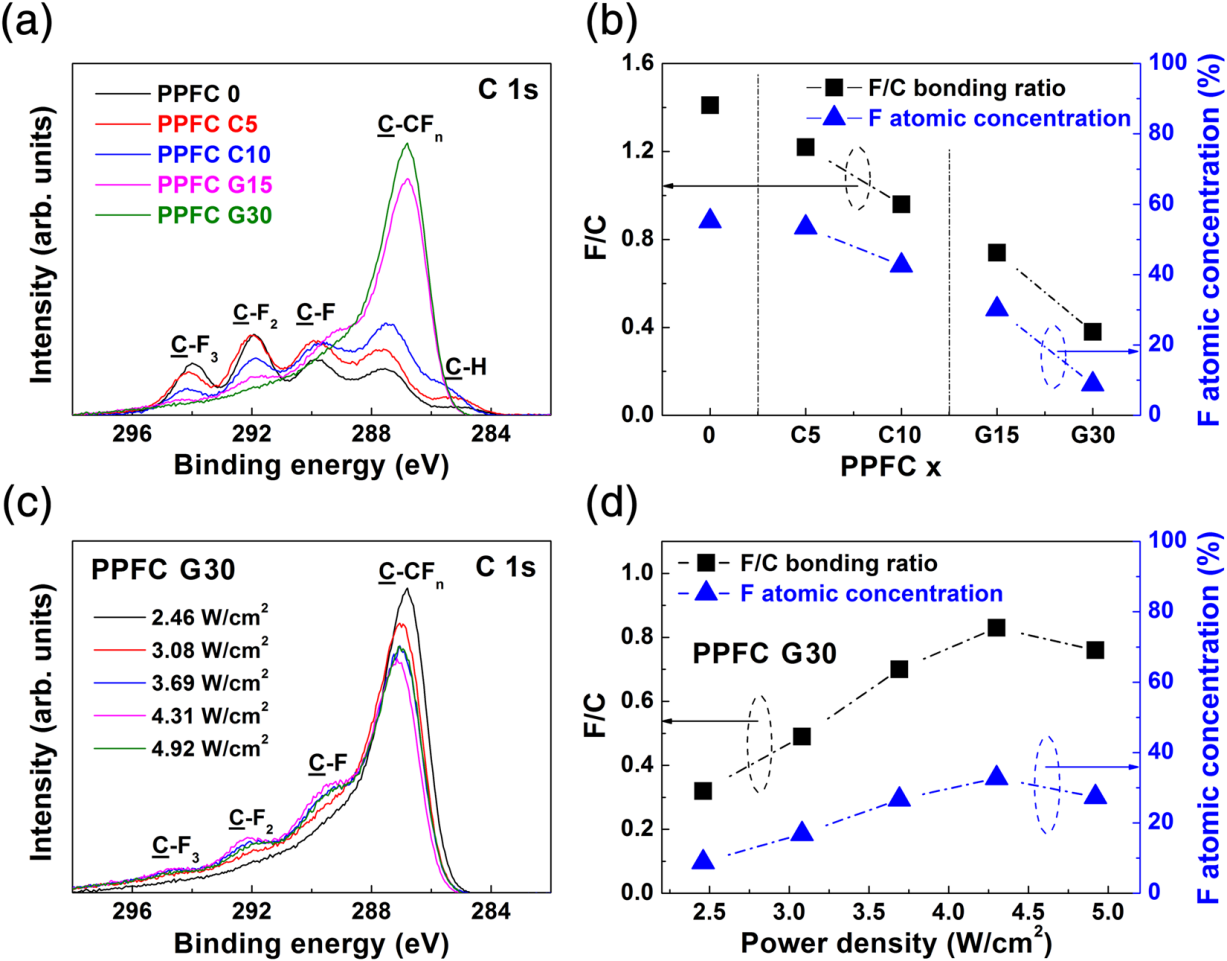
XPS spectra of (**a**) PPFC 0, PPFC C5, PPFC C10, PPFC G15, and PPFC G30 and (**b**) F/C ratio and fluorine atomic concentration calculated by decomposition of XPS C 1*s* peak. XPS spectra of (**c**) PPFC G30 thin films according to sputtering power density and (**d**) F/C ratio and fluorine atomic concentration of PPFC G30 films calculated by decomposition of XPS C 1*s* peak.

**Figure 4:**
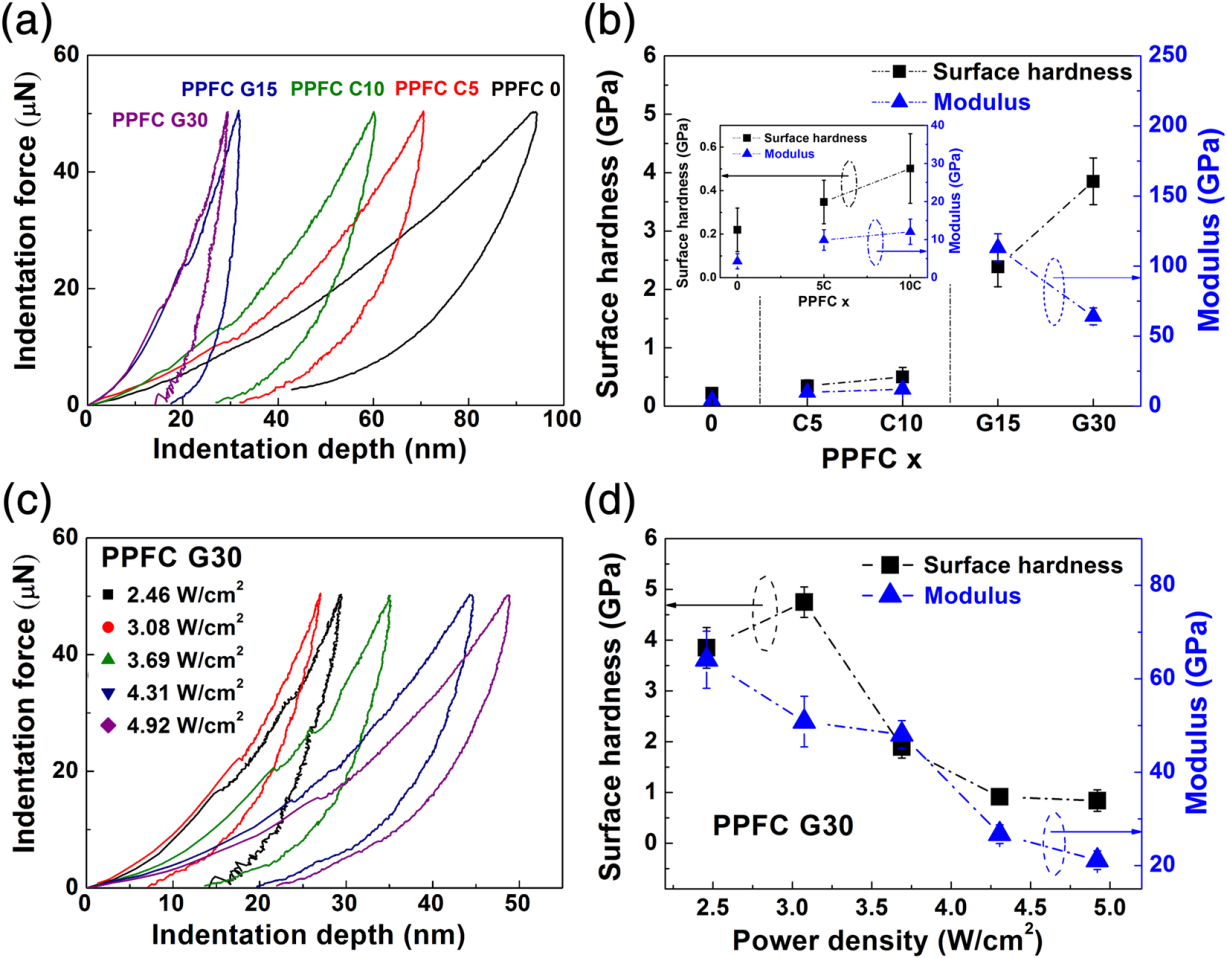
Nanoindentation curves of (**a**) PPFC thin films and (**b**) calculated surface hardness and modulus according to the carbon content of the targets. Nanoindentation curves of (**c**) PPFC G30 thin films according to sputtering power density and (**d**) calculated surface hardness and modulus.

Figure 3(a) shows the XPS spectra of the C 1*s* binding energy regions for the PPFC thin films. In order to remove surface impurities, each sample was subjected to an argon plasma etching treatment for 5s. In some samples, hydrocarbon (C–H) peaks at 285.0 eV remain due to the surface adsorption of organic materials. In this energy region, a binding energy shift occurs by the fluorine atom screening effect near the observed carbon atoms. As reported in previous studies, the peaks at 294.0 eV, 292.0 eV, 239.8 eV, and 286–288 eV represent –CF_3_, –CF_2_-, C-F, and C-CF_n_ carbon crosslinked bonding, respectively^22,25,26,29^. Figure 3(b) shows the fluorine–carbon bond ratios (abbreviated F/C ratios) and F atomic concentrations calculated by the decomposition of the XPS C 1*s* peaks according to the carbon concentrations in the targets. The F/C ratios are calculated by the deconvoluted areas of the –CF_3_, -CF_2_-, and –CF peaks relative to the total area of C 1*s* region^6,30^. The fluorine atomic concentration is quantitatively calculated from the C 1*s* photoelectron in the 284.8 eV region and the F 1*s* photoelectron in the 689.2 eV region, considering atomic scattering factors. The fluorine composition ratio of the PPFC G30 thin film is 8.85%, while that in the PPFC 0 thin film is greatly reduced by 55.17%, because the carbon sputtering yield is two times higher than that of PTFE^28,31^. The C–CF_n_ peak shown in Fig. 4(a) shifts to a lower bonding energy according to the decreasing fluorine atom concentration, also attributed to the decrease of fluorine atoms around carbon atoms. Figure 3(c) shows the XPS spectra of the PPFC G30 films formed with different sputtering power densities; Fig. 3(d) shows the F/C ratio and fluorine atomic concentration calculated by the decomposition of the XPS C 1*s* peak. The atomic concentration of fluorine in the PPFC G30 thin films is increased with sputtering power density, consistent with the optical and surface properties of the PPFC G30 thin films shown in Fig. 2.

The surface hardness values of the thin films were analyzed by the nanoindentation method in order to examine the influence of the composition of the composite target and the sputtering power density on the hardness of the PPFC thin films. Figure 4(a) shows the nanoindentation curve of the PPFC thin films fabricated with varied composite targets. As the carbon composition ratio of the target is increased, the indentation depth of the deposited PPFC thin film decreases, which means that the surface hardness of the PPFC thin film increases. The general formula for calculating the surface hardness through the nanoindentation method is as follows^32^.

1 $${H}_{IT}={P}_{max}/{A}_{c}$$

2 $${A}_{c}=24.5\,{{h}_{c}}^{2}\,({\rm{B}}{\rm{e}}{\rm{r}}{\rm{k}}{\rm{o}}{\rm{v}}{\rm{i}}{\rm{c}}{\rm{h}}\,{\rm{t}}{\rm{i}}{\rm{p}})$$

Here, *H*_*IT*_ is the indentation hardness, *P*_*max*_ is the peak indentation load, and *A*_*c*_ is the projected area of the hardness impression. The *A*_*c*_ for the geometry of the Berkovich tip is given in Eq. (2) from^32^; *h*_*c*_ is the contact height, excluding elastic recovery, at the maximum displacement of the Berkovich tip. Figure 4(b) shows the surface hardness and modulus calculated from the indentation curve. The surface hardness values of the PPFC thin films increase by 17.5 times from 0.22 GPa for PPFC 0 to 3.85 GPa for PPFC G30. This result means that the cross-linking density of the carbon in the PPFC thin films increases, which is consistent with the XPS analysis results. The modulus of the PPFC thin films increases from 4.30 GPa of PPFC 0 to 113.00 GPa of PPFC G15 and then decrease to 64.09 GPa in PPFC G30. This change in modulus indicates that the PPFC G30 thin film is more resilient to external energy, as visually demonstrated by the differences in strain energy dissipation, represented by the areas of the indentation curves for the different films. Figure 4(c) shows the nanoindentation curves of the PPFC G30 thin films according to the sputtering power density; the calculated surface hardness and modulus are shown in Fig. 4(d). The PPFC G30 thin film shows the surface hardness of 4.75 GPa at 3.08 W/cm^2^, which is 22.6 times higher than the 0.22 GPa hardness of PPFC 0; both surface hardness and modulus decrease with increasing power density. This is due to the increase of the fluorine concentration in the PPFC thin films as the sputtering power density is increased and the carbon cross-linking density of the PPFC thin film is decreased, as confirmed by the C-CF_n_ peak shown in Fig. 3(c).

**Figure 5:**
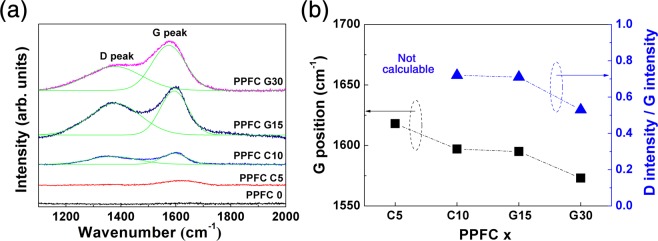
(**a**) Raman spectra of PPFC 0, PPFC C5, PPFC C10, PPFC G15, and PPFC G30 thin films and (**b**) deconvoluted D and G peak positions and relative intensities determined by fitting of the Raman spectra.

Figure 5 shows the results of Raman spectroscopy to further analyze the changes in carbon bonding structures of the PPFC films according to the changing carbon composition ratios of the targets. Figure 5(a) shows the Raman spectra of the PPFC 0, PPFC C5, PPFC C10, PPFC G15, and PPFC G30 thin films. At 1380 cm^−1^, the D peak corresponding to disorder in carbon is detected by the A_1g_ symmetric mode of amorphous carbon; at 1590 cm^−1^, the G peak indicating graphitic carbon is detected by the zone-center E_2g_ symmetric vibration mode of carbon. In the PPFC 0 film, neither D nor G peak is detected. This is the fluorine atom in fluorocarbon films that disrupts the symmetry of the carbon bond and A_1g_ and E_2g_ symmetry, which means that thin films arrangement is changed from a diamond-like to a polymer-like one^33–35^. In the PPFC 5 C thin film, only the G peak is weak at 1612 cm^−1^. Raman curves of PPFC C10, PPFC G15, and PPFC G30 films show equivalent D peaks at 1373 cm^−1^. The G peaks of the PPFC C10, PPFC G15, and PPFC G30 thin films are at 1597 cm^−1^, 1595 cm^−1^, and 1573 cm^−1^, respectively. As the carbon concentration of the PPFC thin films is increased, the intensities of the D peak and G peak tend to increase. This result is consistent with the increasingly intense C-CF_n_ peaks in the XPS analysis shown in Fig. 3(a). Figure 5(b) shows the relationship between the deconvoluted D peak and G peak fitted to the Raman spectra. The ratio of the D/G peak intensity decreases as the carbon concentration in the PPFC is increased, suggesting that PPFC thin films with higher carbon cross-linking densities are formed^23^. The increases in the carbon cross-linking densities in the thin films cause increases in the thin film hardness values. PPFC G30 thin films fabricated at high sputtering power densities show decreases in D and G peak intensity; at the power higher than 3.69 W/cm^2^, the D and G peaks are not detected, because the A_1g_ and E_2g_ symmetry are collapsed by the increased fluorine content. The XPS and Raman spectral analyses show that the number of bonds between carbons increases as the carbon concentration in the target is increased, and that carbon cross-linking density becomes high in the PPFC thin films.

The PPFC G30 thin film deposited at 3.08 W/cm^2^ showed a high surface hardness of 4.75 GPa, but the fluorine atomic fraction was lower than PPFC 0, as reflected by the optical transmittance of 85.83% (5.77% lower than that of PPFC 0) and the surface energy (water contact angle) of 30.95 × 10^−3^ N/m (80°), which is 21.22 × 10^−3^ N/m higher than that of PPFC 0. The PPFC G30 film with a power density of 4.92 W/cm^2^ showed a twofold increase in the fluorine composition ratio relative to that of the film formed at 3.08 W/cm^2^, a light transmittance of 91.32%, and a surface energy (water contact angle) of 15.99 × 10^−3^ N/m (100°). On the other hand, the surface hardness of the highest power density sputtered PPFC G30 thin film 4.92 W/cm^2^ was 0.91 GPa, which is one-fifth of PPFC deposited at G30 3.08 W/cm^2^, but four times higher than the 0.22 GPa hardness measured from PPFC 0. The PPFC thin films prepared by the MF sputtering of composite polymer targets, as proposed in this study, show controllable physical properties of optical transmittance, surface hardness and surface water repellency with manipulation of the composition of the target and the sputtering power density.

## Conclusion

In this study, CNT/PTFE and Graphite/PTFE composite targets were fabricated and PPFC thin films were deposited by MF sputtering using the targets. The PPFC 0, PPFC C5, and PPFC C10 thin films with lower carbon concentrations in the film matrix backbones showed high optical transmittances and surface water repellency irrespective of sputtering power, while the PPFC G15 and PPFC G30 films with higher carbon concentrations showed lower optical transmittance and surface water repellency at the sputtering power density of 2.46 W/cm^2^. The XPS and Raman spectroscopic analyses of the PPFC thin films revealed that, as the carbon concentration in target was increased, the carbon cross-linking density of the PPFC thin films increased, yielding a hardness value 21.6 times that of the PPFC 0 thin films. As the sputtering power density was increased, the fluorine concentration of the PPFC thin film increased, as indicated by XPS analysis. As the fluorine concentration increased, the optical transmittances and water repellency of the thin films increased and the carbon cross-linking densities decreased. In this study, high-hardness PPFC thin films were successfully fabricated by using polymer composite targets, and it was confirmed that the composition and carbon cross-linking density of the thin films could be controlled by changing the sputtering power density.

## Methods

### Preparation of carbon/PTFE composite target

To investigate the effect of carbon contents on PPFC thin films, we designed polymeric composite target mixed with carbon materials. Multiwall CNT powder (HANOS CM-280, Hanwha Chemical) and PTFE powder (A7-J, Dupont Mitsui) were mixed in weight ratios of 5:85, 10:90, and 15:85 to fabricate relatively low carbon contents target. Graphite powder (TIMREX® KS44, Timcal) and PTFE powder were also mixed at weight ratios of 15:85, and 30:70 to fabricate composite targets having a higher carbon contents than CNT/PTFE. Each pre-mixed powder was press-molded with heat treatment and then milled to form a disk type target of 10 cm in radius and 6 mm in height. Pressure of compression was 200 kgf/cm^2^ and heat treatment temperature was 370 °C. The CNT/PTFE composite targets are named CNT 5, CNT 10 and CNT 15. The Graphite/PTFE composite targets are named Graphite 15, and Graphite 30.

### Carbon-rich PPFC thin film fabrication

We designed the vacuum chamber (Cluster Sputter system, Charmtron) with a rotatable substrate size of 200 × 200 mm^2^. The sputter guns on the opposite side of the substrate were composed of two sputtering cathodes apparatus. Target-to-substrate distance was fixed at 240 mm. The sputtering chamber was evacuated by a rotary vane pump and cryogenic pump. Refrigerated surfaces at 10 K in cryogenic pump trapped residual gas to a level of 6 × 10^−4^ Pa. The processing atmosphere was 0.93 Pa argon gas controlled by a mass flow controller using throttle valve system. CNT 5, CNT 10, CNT 15, Graphite 15 and Graphite 30 composite targets were sputtered with a 40 kHz MF power source (PE II, Advanced Energy). In sputtering process, dual magnetron cathodes was used to improve sputtering efficiency. Thin films were deposited at power densities within 0.62~4.92 W/cm^2^. The 100 nm carbon-rich PPFC films were deposited on a 188 μm thick of PET film (Kimoto) and a silicon substrate (silicon (100) wafer, iTASCO).

### Characterizations of carbon-rich PPFC thin films

The optical properties of the PPFC thin films were analyzed using a spectrophotometer (U-4100, Hitachi) in the wavelength range 300–2400 nm and compared in terms of transmittance at 550 nm and the b* parameter of yellowness. Surface properties of the PPFC thin film were measured by a contact angle analyzer (Phoenix 300 Touch, Surface Electro Optics). 2 μL of water was used as the contact angle measurement liquid, and its surface tension was 72 × 10^−3^ N/m. The surface energies were calculated by the Girifalco–Good–Fowkes–Young method. The chemical structures were analyzed using Raman spectroscopy (inVia Raman Microscope, Renishaw) and X-ray photoelectron spectroscopy (XPS, AXIS NOVA, Kratos). The surface hardness and modulus values were measured using the nanoindentation method (Nanoindentation, Anton Paar). The hardness measurements at an indentation force of 50 μN were analyzed.

## Supplementary information

Supplementary Information

## References

[1] Biederman, H. *Plasma Polymer Films* 13–24 (World Scientific, 2004).

[2] Biederman H (2000). RF sputtering of polymers and its potential application. Vacuum.

[3] Biederman H (2001). RF magnetron sputtering of polytetrafluoroethylene under various conditions. Thin Solid Films.

[4] Zaporojtchenko V, Podschun R, Schürmann U, Kulkarni A, Faupel F (2006). Physico-chemical and antimicrobial properties of cosputtered Ag-Au/PTFE nanocomposite coatings. Nanotechnology.

[5] Schürmann U, Hartung W, Takele H, Zaporojtchenko V, Faupel F (2005). Controlled syntheses of Ag-polytetrafluoroethylene nanocomposite thin films by co-sputtering from two magnetron sources. Nanotechnology.

[6] Iwamori S (2007). Adhesion and tribological properties of sputtered polymer thin films with thermally stable polymer targets. J. Vac. Soc. Jpn..

[7] Iwamori S, Noda K (2012). Optical property of fluorocarbon thin films deposited onto polyester film substrate by an RF sputtering. Mater. Lett..

[8] Gonon P, Sylvestre A (2002). Dielectric properties of fluorocarbon thin films deposited by radio frequency sputtering of polytetrafluoroethylene. J. Appl. Phys..

[9] Sarkar DK, Farzaneh M, Paynter RW (2008). Superhydrophobic properties of ultrathin RF-sputtered Teflon films coated etched aluminum surfaces. Mater. Lett..

[10] Kamegawa T, Shimizu Y, Yamashita H (2012). Superhydrophobic surfaces with photocatalytic self-cleaning properties by nanocomposite coating of TiO_2_ and polytetrafluoroethylene. Adv. Mater..

[11] Takashi K, Koichi I, Hiromi Y (2017). Multifunctional surface designed by nanocomposite coating of polytetrafluoroethylene and TiO_2_ photocatalyst self-cleaning and superhydrophobicity. Sci. Rep..

[12] Huang C, Pan C-H, Tsai C-Y, Tseng I-Y (2013). Fabrication of oleophobic fluorocarbon film by 13.56 MHz CH_2_F_2_/Ar plasma chemical vapor deposition. Surf. Coat. Tech..

[13] Tang G, Ma X, Sun M, Li X (2005). Mechanical characterization of ultra-thin fluorocarbon films deposited by RF magnetron sputtering. Carbon.

[14] Jiang X (2014). Improvement of adhesion strength and scratch resistance of fluorocarbon thin films by cryogenic treatment. Appl. Surf. Sci..

[15] Jin JH, Ko J-H, Yang SC, Bae B-S (2010). Rollable transparent glass-fabric reinforced composite substrate for flexible devices. Adv. Mater..

[16] Gustafsson G (1992). Flexible light-emitting diodes made from soluble conducting polymers. Nature.

[17] Li L, Jones PM, Hsia Y-T (2011). Characterization of a nanometer-thick sputtered polytetrafluoroethylene film. Appl. Surf. Sci..

[18] He X-M, Hakovirta M, Nastasi M (2005). Hardness, hydrophobic and optical properties of fluorine and boron co-alloyed diamond-like carbon films. Mater. lett..

[19] Butter RS, Waterman DR, Lettington AH, Ramos RT, Fordham EJ (1997). Production and wetting properties of fluorinated diamond-like carbon coatings. Thin Solid Films.

[20] Kim SH, Kim M, Lee JH, Lee S-J (2018). Self-cleaning transparent heat mirror with a plasma polymer fluorocarbon thin film fabricated by a continuous roll-to-roll sputtering process. ACS Appl. Mater. Interfaces.

[21] Newman SB, Young RW (1967). Indentation hardness of the fingernail. J. Invest. Dermatol..

[22] Kim SH (2017). Fluorocarbon thin films fabricated using carbon nanotube/polytetrafluoro ethylene composite polymer targets via mid-frequency sputtering. Sci. Rep..

[23] Robertson J (2002). Diamond like amorphous carbon. Mater. Sci. Eng. R.

[24] Li QY, Wang FM, Zhang L (2013). Study of colors of diamond-like carbon films. Sci. China Phys. Mech. Astron..

[25] Yamada Y, Kurobe T (1993). X-ray photoelectron spectroscopy of fluorocarbon films deposited by RF sputtering. Jpn. J. Appl. Phys..

[26] Kousal J (2005). RF magnetron sputtering and evaporation of polyisobutylene and low density polyethylene. Surf. Coat. Technol..

[27] Mathias E, Muller CH (1967). The decomposition of polytetrafluoroethylene in a glow discharge. J. Phys. Chem..

[28] Rzeznik L, Fleming Y, Wirtz T, Philipp P (2016). Experimental and simulation-based investigation of He, Ne and Ar irradiation of polymers for ion microscopy. Beilstein J. Nanotech..

[29] Ueda A (2009). Fabrication of electrochemically stable fluorinated nano-carbon film compared with other fluorinated carbon materials. Carbon.

[30] Stelmashuk V, Biederman H, Slavínská D, Zemek J, Trchová M (2005). Plasma polymer films RF sputtered from PTFE under various argon pressures. Vacuum.

[31] Kolasinski RD, Polk JE, Goebel D, Johnson LK (2008). Carbon sputtering yield measurements at grazing incidence. Appl. Surf. Sci..

[32] Jee AY, Lee MY (2010). Comparative analysis on the nanoindentation of polymers using atomic force microscopy. Polymer Testing.

[33] Jacobsohn LG, Maia da Costa MEH, Trava-Airoldi VJ, Freire FL (2003). Hard amorphous carbon–fluorine films deposited by PECVD using C_2_H_2_–CF_4_ gas mixtures as precursor atmospheres. Diam. Relat. Mater..

[34] d’Agostino R, Lamendola R, Favia P, Giquel A (1994). Fluorinated diamond like carbon films deposited from radiofrequency glow discharge in a triode reactor. J. Vac. Sci. Technol. A.

[35] Touhara H, Okino F (2000). Property control of carbon materials by fluorination. Carbon.

