# Supplementary material for: Effects of carbon concentration on high-hardness plasma-polymer-fluorocarbon film deposited by mid-range frequency sputtering
Source: Sci Rep. 2019 Jul 23;9:10664. doi: 10.1038/s41598-019-46993-0 (PMC6650446; doi:10.1038/s41598-019-46993-0)
Supplement: Supplementary file 1 — Supplementary Information [file 41598_2019_46993_MOESM1_ESM.doc]

**Supplementary Information**

**Effects of carbon concentration on high-hardness plasma-polymer-fluorocarbon film deposited by mid-range frequency sputtering**

Sung Hyun Kim, Mac Kim, Min Seop Um, Woo Jin Choi, Jae Heung Lee, Yong Suk Yang^*^ and Sang-Jin Lee^*^

Sung Hyun Kim, Mac Kim, Min Seop Um, Woo Jin Choi, Jae Heung Lee, Sang-Jin Lee

Chemical Materials Solutions Center, Korea Research Institute of Chemical Technology, Daejeon 34114, Korea

Sung Hyun Kim, Yong Suk Yang,

Department of Nano Fusion Technology, Pusan National University, Busan 46241, Korea

* [ysyang@pusan.ac.kr](mailto:*ysyang@pusan.ac.kr), * [leesj@krict.re.kr](mailto:leesj@krict.re.kr) (co-corresponding)

**Supplementary Figures**


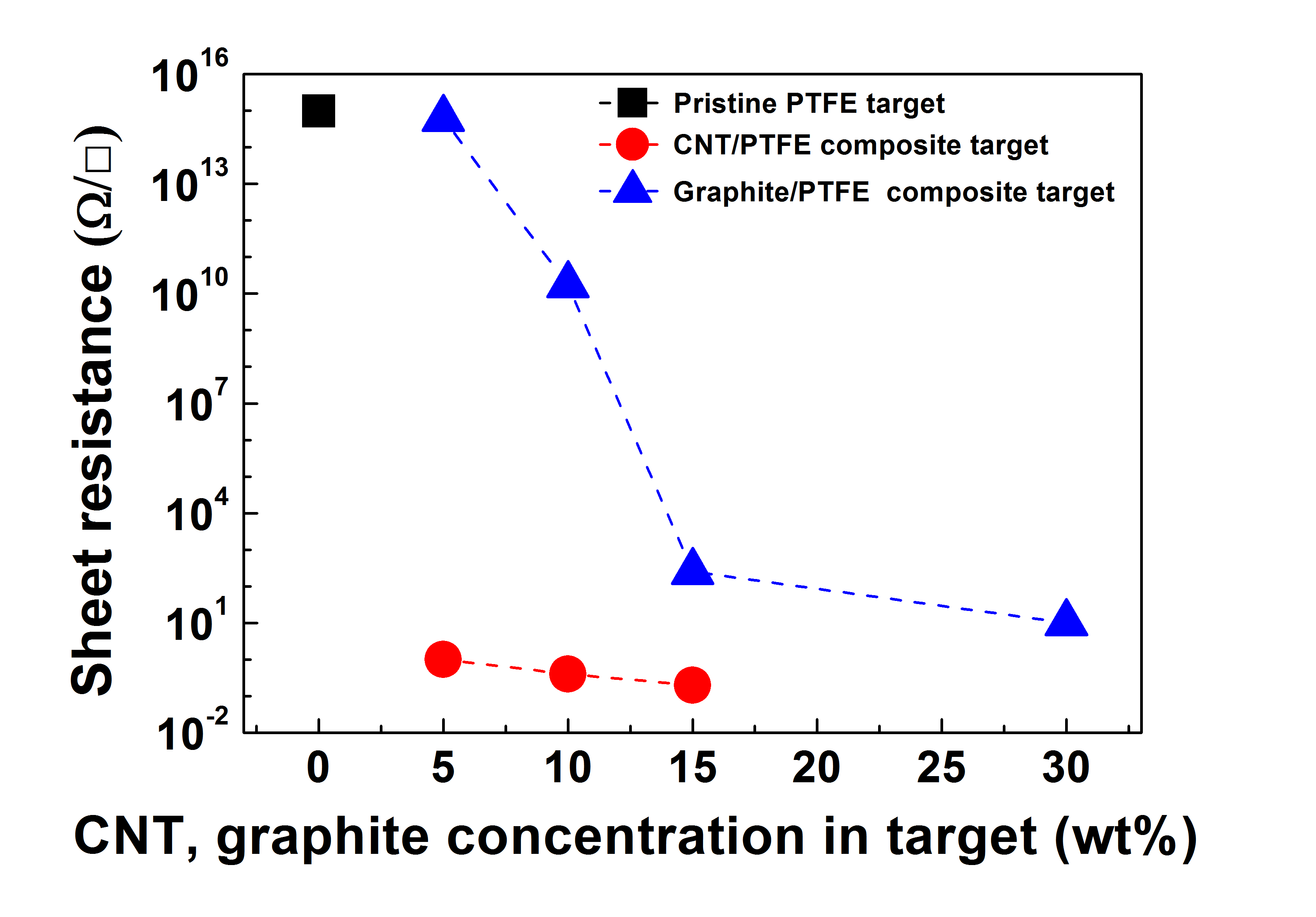


**Supplementary Figure S1.** Surface resistances of composite targets according to composition ratios of CNT and graphite.


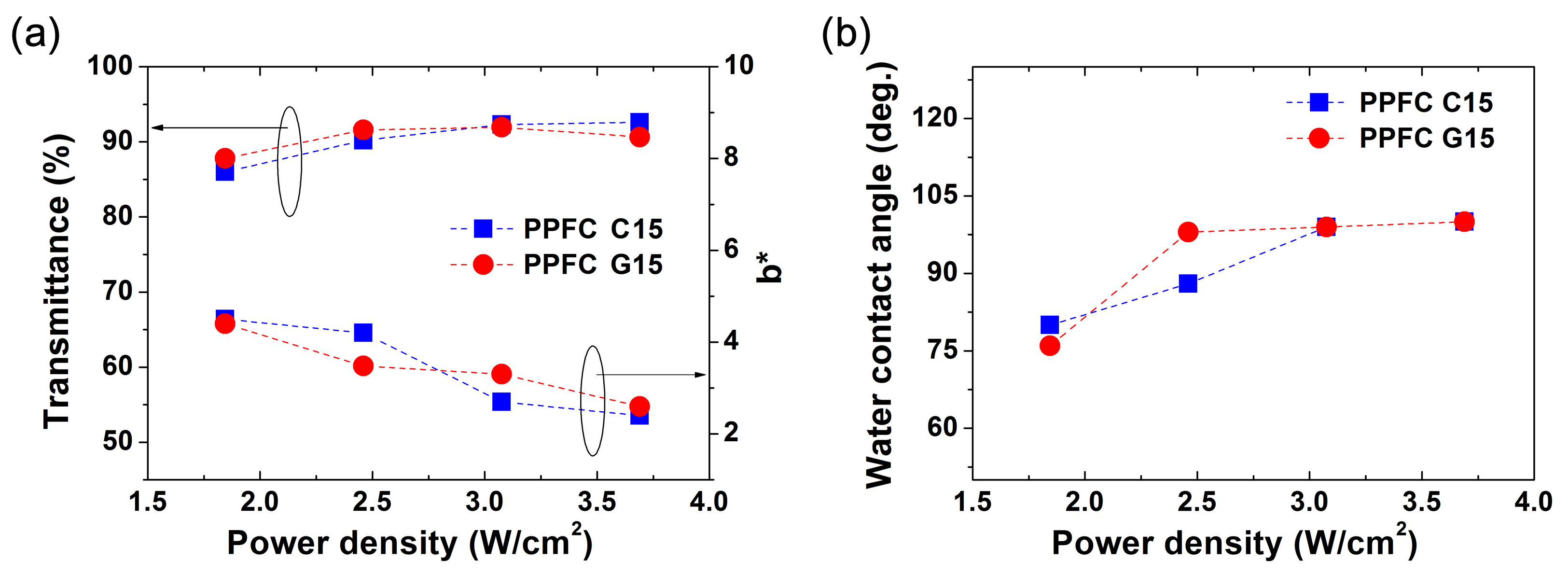


**Supplementary Figure S2.** (a) Optical transmittances and *b** values and (b) water contact angles according to sputtering power densities of PPFC C15 and PPFC G15 thin films.
